# High-contrast switching and high-efficiency extracting for spontaneous emission based on tunable gap surface plasmon

**DOI:** 10.1038/s41598-018-29624-y

**Published:** 2018-07-26

**Authors:** He Hao, Juanjuan Ren, Xueke Duan, Guowei Lu, Iam Choon Khoo, Qihuang Gong, Ying Gu

**Affiliations:** 1 State Key Laboratory for Mesoscopic Physics, Collaborative Innovation Center of Quantum Matter, School of Physics, Peking University, Beijing, 100871 China; 2 Collaborative Innovation Center of Extreme Optics, Shanxi University, Taiyuan, Shanxi 020006 China; 3 Department of Electrical Engineering, 121 Electrical Engineering East, Pennsylvania State University, University Park, Pennsylvania, PA 16802 USA

## Abstract

Controlling spontaneous emission at optical scale lies in the heart of ultracompact quantum photonic devices, such as on-chip single photon sources, nanolasers and nanophotonic detectors. However, achiving a large modulation of fluorescence intensity and guiding the emitted photons into low-loss nanophotonic structures remain rather challenging issue. Here, using the liquid crystal-tuned gap surface plasmon, we theoretically demonstrate both a high-contrast switching of the spontaneous emission and high-efficiency extraction of the photons with a specially-designed tunable surface plasmon nanostructures. Through varying the refractive index of liquid crystal, the local electromagnetic field of the gap surface plasmon can be greatly modulated, thereby leading to the swithching of the spontaneous emission of the emitter placed at the nanoscale gap. By optimizing the material and geometrical parameters, the total decay rate can be changed from 103γ_0_ to 8750γ_0_, [γ_0_ is the spontaneous emission rate in vacuum] with the contrast ratio of 85. Further more, in the design also enables propagation of the emitted photons along the low-loss phase-matched nanofibers with a collection efficiency of more than 40%. The proposal provides a novel mechanism for simultaneously switching and extracting the spontaneous emitted photons in hybrid photonic nanostructures, propelling the implementation in on-chip tunable quantum devices.

## Introduction

Controlling spontaneous emission (SE) at subwavelength scale is of fundamental and practical importance in nanophotonics, cavity quantum electrodynimics, and quantum information processing, propelling the performance of on-chip quantum devices such as single photon sources^1–3^, nanolasers^4,5^, and nanophotonic detectors^6,7^. In 1946, Purcell predicated that the lifetime of a quantum emitter is not an intrinsic parameter but a function of photonic density of states where the emitter resides^8^. Since then, the modified SE rate has been observed in various photonic structures, such as microcavities^9^, wisperirng guided cavities^10,11^, photonic crystals^12,13^, and so on. In recent years, large enhancement of SE have also been reported in theoretically and experimental studies of plasmonic metamaterials^14,15^ and plasmonic nanostructures^16–23^; sepcifically, in gap surface plasmon (GSP) structures, the emission rate can exceed 1000*γ*_0_, where *γ*_0_ is the SE rate in vacuum, due to the presence of ultrastrong hotspots^18–23^. It is important to note here that these plasmon nanostructures are passive; once fabricated, they do not allow tunability demanded by state-of-the-art nanophotonic devices.

Several approaches have also been developed for modulation of the SE^24–34^. One way of controlling SE rate is by means of the Auger processes. For examples, by incorporating quantum dots in an electrically control p-i-n dipole maintained at liquid-nitrogen temperature, the energy relaxation into electron-hole pairs can be tuned electrically, thereby the switching of SE is attained^24,25^; by combining neutral nitrogen-vacancy centre with a novel diamond diode^26,27^ or through replacing the semiconductor quantum dots with organic molecules^28^, room-temperature electrical modulating SE can be realized. Another more obvious way of controlling SE is by changing the optical mode density. By placing the quantum dots on top of 2D material such as graphene^29–31^ or MoS_2_^32,33^, which permits actively controlling of the energy flow from emitters into optical or plasmonic excitations, the emission intensity can be changed by several folds through tuning the Fermi level of the 2D materials. The SE rate can also be tuned by introducing reconfigurable multiscale biological material into quantum dot-nanoparticle system^34^, where the distance between quantum dot and nanoparticle can be controlled and in turn influences the lifetime of quantum dot about two folds. Besides tunability, another highly desirable feature is efficient coupling of the emitted photons into some waveguide nanostructures^35–37^.

**Figure 1:**
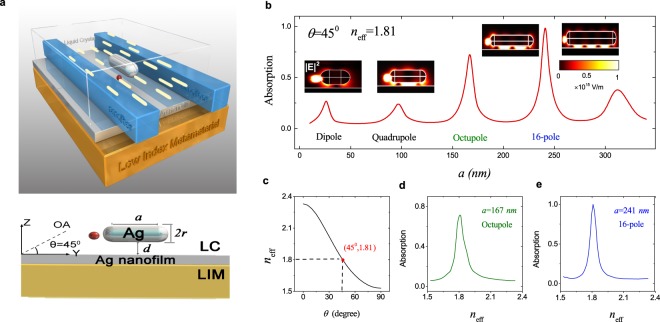
(**a**) Schematic of the hybrid tunable GSP nanostructure. A silver nanorod is coupled to a silver nanofilm cladded with LC and LIM. The single dipole emitter (red dot) is embedded in the nanogap and double low-loss nanofibers are symmetrically positioned on the nanofilm to efficiently route the photons.The GSPs of the designed hybrid system. (**b**) The absorption spectra of the GSPs with varying the length *a* of Ag nanorod. Here *λ* = 720 *nm*, *d* = 10 *nm*, and a silver nanofilm of 50 *nm* is sandwiched between the LC with *n*_o_ = 1.53, *n*_e_ = 2.33, Δ*n* = 0.8^54^ and LIM with *n*_d_ = 0.4 + 0.05 *i*^55^. For *θ* = 45°, *n*_eff_ = 1.81, with increasing the length of nanorod, its dipole, quadrupole, octupole, and 16-pole GSPs ordinally appear at *a* = 26, 90, 167, and 241 *nm* respectively when the dipole emitter is placed near the left end of the nanorod. For the different kind of GSPs, the hotspots appear at the nanoscale gap with different number and symmetry. (**c**) The effective refractive index *n*_eff_ of LC with varying *θ* for *n*_o_ = 1.53, *n*_e_ = 2.33. The absorption spectra of (**d**) octupole and (**e**) 16-pole modes as a function of *n*_eff_ with the same parameters as those in (**b**).

Despite these progresses made in enhanced and switchable spontaneous emission, there is no approach/design capable of simultaneously achieving high modulation of fluorescence intensity and efficient coupling of the emitted photons into low-loss nanophotonic structures. In this paper, we theoretically demonstrate high-contrast switching of the spontaneous emission and high-efficiency extraction of the emitting photons with a tunable surface plasmon nanostructure containing liquid crystal and two dielectric nanofibers (Fig. 1a). Through varying the refractive index of the liquid crystal, the local electromagnetic field of the gap surface plasmon can be greatly modulated to influence the spontaneous emission while the suitably placed nanofibers enable efficient extraction of the emitted photons.

It is known that the liquid crystal (LC) can be used to modulate the surface plasmon polariton, where mode transformation and recombination occur with varying its permittivity tensor^38^. Accordingly, the change of its density of optical modes gives rise to modulation of the SE rate. However, the largest modulation can only reach 2.5 folds in the planar plasmon nanostructure^39^, which do not meet the application requirement of on-chip quantum devices. Here, we exploit the fact that when the metallic nanorod is very close to the metallic nanofilm, the existence of gap surface plasmon can be effectively controlled through varying the permittivity tensor of the LC. Thus, if the quantum emitter is suited at the hot spots inside the nanoscale gap, its SE rate will be greatly modulated; the emitted photons could then be effectively extracted by the phase-matched low-loss nanofibres.

For practical use of emitted photons, it is necessary to obtain a significant contrast ratio as well as large enhancement of the SE. By optimizing the material parameters, the SE rate of dipole emitter can be modulated from 103*γ*_0_ to 8750*γ*_0_, i.e., a high-contrast ratio of 85. Most notably, with suitably dye-doping, it only takes several nanoseconds^40^ to change the effective refractive index of the LC to realize a sizeable contrast ratio of ∼10; this means that the operation time of SE switching can be compressed into nanosecond level. Additionally, for practical applications in on-chip photonic circuits, the phase-matched dielectric nanofibers are capable of collecting and routing the photons with an efficiency of more than 40%.

This design thus provides the flexible and integrated platform for simultaneously switching and routing the spontaneously emitted photons, a highly desirable feature in the development of nanolaser^4,5^, single photon source^1–3^, nanostructure-based cavity quantum electrodynamics^41,42^, and the quantum information science^43^. If the switchable decay rate can be combined with an optical resonator, the threshold of nanolasers or spacers^4,5^ will thus possess the tunable properties for the optimized utilization of the pump power. In the case where only a single emitter is present, the resulting switching of single photons could provide the basis for controllable single photon sources^1–3^. The presence and absence of photons can map the on and off state, generating a Schrodinger-cat state^44^, functionalizing a quantum memory^45^, or building a quantum-logic gate^46^. One can also envision employing the continuous modulation of the decay rates and the guided photons with high collection efficiency for on-chip quantum devices, such as quantum repeaters^47^ and single-photon router^48^.

## Results

### Design of the hybrid tunable GSP structure

We consider the hybrid tunable GSP system as shown in Fig. 1a, where a silver nanorod is closely coupled to a silver nanofilm cladded with the LC and low index metamaterial (LIM) and two low-loss nanofibers are symmetrically positioned on the nanofilm to efficiently route the emitted photons. The nanoscale gap between metallic nanorod and nanofilm guarantees the existence of the GSP^49,50^, whose hotspots lead to the large enhancement of the SE^19–23^. Through varying the refractive index of the LC by various means^40,51^ to modulate the local electromagnetic field of the GSP, the SE rate of the emitter placed at the nanoscale gap is modulated. Without the Ag nanorod above the plasmon nanofilm, the SE rate of the quantum emitter embedded into the LC can be tuned only about 2.5 times^39^. The role of the LIM is to further enhance the SE rate since it almost has not any effect on modulation of the decay rate^39,52^. Additionally, as shown in Fig. 1a, two low-loss phase-matched nanofibers are symmetrically positioned above the nanofilm to efficiently collect and route the emitted photons, leading to the direct use in on-chip quantum devices. As detailed in the following, our design has the capability of simultaneously realizing high-contrast switching and high-efficiency routing of the spontaneously emitted photons.

It is known that in this GSP nanostructure, the total decay rate γ_total_ of a quantum emitter embedded in the gap comes from three channels^23^: the decay rate γ_spp_ into the surface plasmon channel, the radiative decay rate γ_r_ into free space, and the decay rate γ_nr_ into nonradiative channels, where the absorption of both silver nanorod and its image in the nanofilm made contributions to γ_nr_. Compared with the GSP nanostructure designed in ref.^21^ in the present structure, the presence of LC provides excellent tunable properties of the SE.

**Figure 2:**
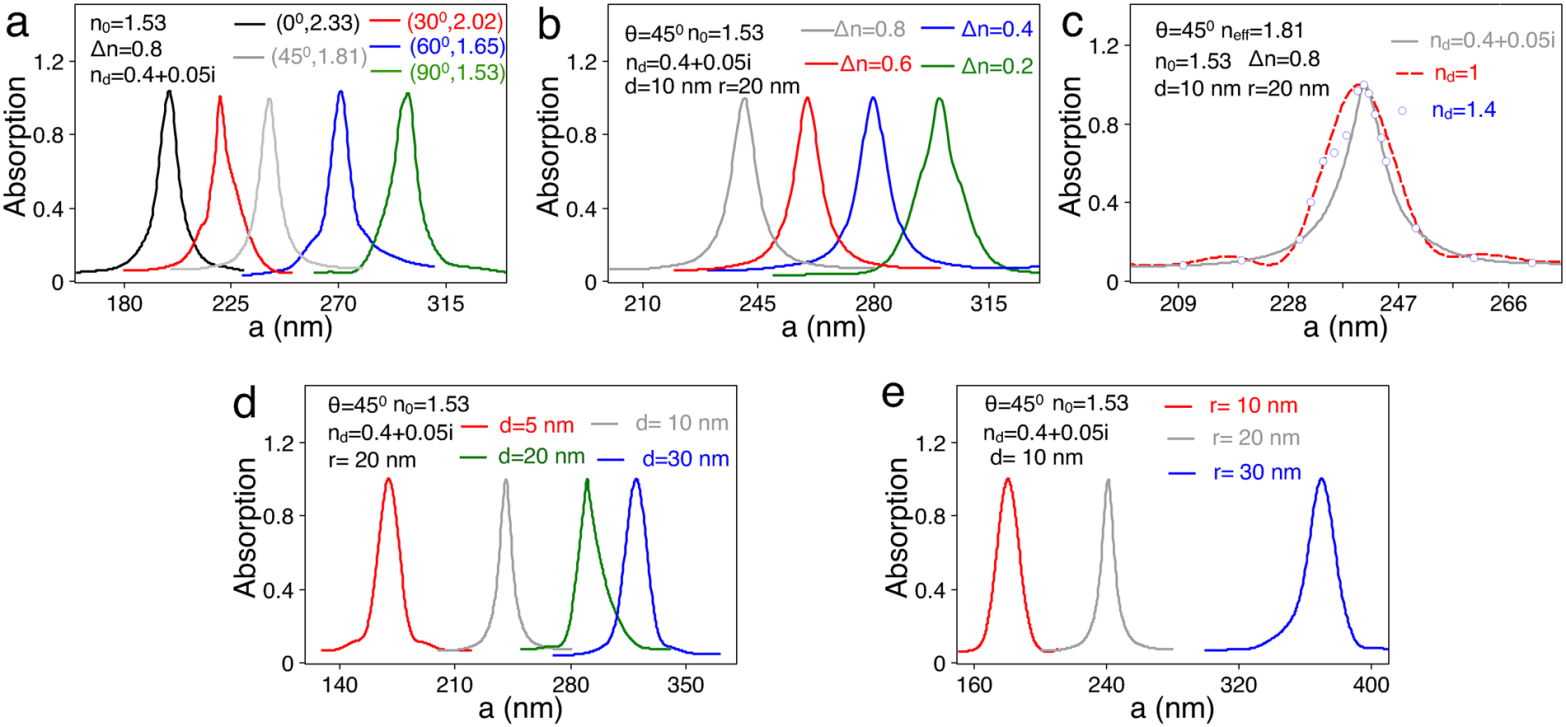
The effects of material and geometric parameters on the GSPs. (**a**–**c**) The absorption spectra for the 16-pole mode with varying (*θ*, *n*_eff_), Δ*n* and *n*_d_. The geometric parameters are the same as those in the Fig. 1b. (**d**,**e**) The absorption spectra for the 16-pole mode with varying *d* and *r*. The material parameters are the same as those in the Fig. 1b.

To obtain these decay rates, 3D finite element simulations are performed with commercial COMSOL Multiphysics software(See Supplementary Information). The module dimension is chosen to correspond with the behavior of an infinitely extended metal film and the dielectric constant of metal is taken from experimental data^53^. The quantum emitter located at the nanogap is simulated by a classical electrical dipole, with an emission wavelength of *λ* = 720 *nm* that provides maximum spectral overlap with gap plasmon. To achieve the largest enhancement of the decay rate and highest contrast ratio, we select the combination of Ag nanorod (composed of a cylinder of length a and two semispheres with radius *r* = 20 *nm*) and Ag nanofilm from the sets of Ag(Au) nanorod and Ag(Au) nanofilm. In the designed hybrid system, reducing the thickness of silver nanofilm can accelerate the SE rate, thus we set the thickness of the nanofilm at 50 *nm*. The strength of the hotspot relies heavily on the distance *d* between nanorod and nanofilm^22,49,50^. In order to obtain brighter hotspot, we set the gap size as small as 10 *nm*. For the higher contrast ratio, the optical axis of LC is set in the perpendicular plane (YZ plane) and makes an angle *θ* with respect to the Y axis, as shown in Fig. 2(a). Under such assumption, the permittivity tensor of LC can be expressed as follows:

$$\hat{\varepsilon }=[\begin{array}{ccc}{n}_{o}^{2} & 0 & 0\\ 0 & {n}_{e}^{2}co{s}^{2}\theta +{n}_{o}^{2}si{n}^{2}\theta  & ({n}_{o}^{2}-{n}_{e}^{2})sin\theta cos\theta \\ 0 & ({n}_{o}^{2}-{n}_{e}^{2})sin\theta cos\theta  & {n}_{o}^{2}co{s}^{2}\theta +{n}_{e}^{2}si{n}^{2}\theta \end{array}]$$

Generally, the effective index of LC ${n}_{{\rm{eff}}}={n}_{o}{n}_{e}/\sqrt{{n}_{e}^{2}si{n}^{2}\theta +{n}_{o}^{2}co{s}^{2}\theta }$. It is important to note that these material parameters used here have been reported recently^54,55^, suggesting that this system can indeed be possibly fabricated in the laboratory.

### Optical mode analysis of tunable GSP system

The density of the optical modes plays a center role in changing the decay rate of the quantum emitter^8,23^. Here, the dominant mode is the GSP, which can be intuitively looked as the coupled mode of metallic nanorod and nanofilm with a nanoscale gap^23,49,50^. The highly concentrated electromagnetical field in the gap, i.e., the hotspot, leads to a large SE enhancement of the quantum emitter^19–23^. In the following, we focus on the analysis of the GSP with the variation of the length of Ag nanorod, the orientation of the optical axis of the LC, and the refractive indexes of the LIM, which are the important factors to obtain the large SE rate and modulation depth. The double dielectric low-loss nanofibers here have only a very small contribution to the GSPs and the SE rates, so in the mode analysis and the switching of the SE, it is reasonable to ignore their effects (See Supplementary Information).

Figure 1b displays the absorption spectra of the GSPs with the length of Ag nanorod ranging from 5 *nm* to 350 *nm* under the specific optical axis of *θ* = 45°. A single dipole is placed at the end of nanorod to guarantee that all possible modes can be excited efficiently. Here *λ* = 720 *nm*, *d* = 10 *nm*, and a silver nanofilm of 50 *nm* is sandwiched between the LC with *n*_o_ = 1.53, *n*_e_ = 2.33, Δ*n* = 0.8^54^ and LIM with *n*_d_ = 0.4 + 0.05 *i*^55^. Considering the case of *θ* = 45°, *n*_eff_ = 1.81, for the lowest mode, i.e., the dipole mode, its resonance length is *a* = 26 *nm*, and it has odd symmetry with two hotspots^23,49,50^. With increasing the nanorod length, multipole GSPs appear, and are manifested by the increase in the number of hotspots in the flat region of the gap^56^, c.f. the insert pictures in Fig. 1b. In particular, the quadrupole mode resonates at *a* = 96 *nm* depicts three hotspots, among which the brightest one is at the middle of the nanogap. When the length of rod increases to *a* = 167 *nm*, octupole mode is excited with two bright hotspots and two weak ones. For *a* = 241 *nm* the 16-pole mode appears with three bright hotspots and two weak ones.

Generally speaking, for a particular kind of the GSP, the brighter hotspot produces more enhancement of the decay rates^23^, also GSPs with narrower spectra provide better tunable property of the SE. By comparing the linewidth of multipole GSPs, octupole with narrow spectrum and 16-pole modes are selected to be used for modulation of the SE. In the following, we will focus on these two modes. The mechanism of modulating SE is to first change the effective refractive index *n*_eff_ of the LC to tune the GSP. Therefore, the dependence of *n*_eff_ on *θ* is first illustrated (Fig. 1c). It is seen that *n*_eff_ can be changed from 1.53 to 2.33 when *θ* is varied from 0° to 90°. The resulting changes in the lineshape for the octupole and 16-pole GSPs are shown in Fig. 1d and e. It is found that the GSP peak appears and disappears with varying *n*_eff_ or *θ*; the resultant changes in the local field of the nanoscale gap or the density of the optical modes causes the modulation of the decay rate.

Figure 2a displays the variation of the resonance length *a* for the 16-pole mode with changes in *n*_eff_ or the optical axis *θ*. With the decrease of *n*_eff_, the resonance length shifts to the longer value; when *n*_eff_ = 2.33 (*θ* = 0°), for example, the resonance length *a* = 200 *nm* whereas for *n*_eff_ = 1.53 (*θ* = 90°), *a* = 300 *nm*. Besides the optical axis *θ*, the effective refractive index of the LC can be changed by a variety of mechanisms such as temperature, electricity, or optics^40,51^. The resonance length *a* also strongly depends on the birefringence Δ*n*. As shown in Fig. 2b, when Δ*n* = 0.8, *a* = 241 *nm*; but for Δ*n* = 0.2, it increases to *a* = 300 *nm*. Note that the refractive index *n*_d_ of LIM has little influence on the resonance length. Figure 2c shows that *a* ≈ 241 *nm* whether one uses the LIM with *n*_d_ = 0.4 + 0.05 *i* or conventional material with *n*_d_ = 1.0 or *n*_d_ = 1.4. In the following, we will see that the enhancement of the SE rate is determined by *n*_d_^39^, but its modulation range or the contrast ratio of the switching is determined by Δ*n* and *n*_eff_.

The properties of the GSPs are understandably geometry-dependent. The data shown in Fig. 2d illustrate the trend of absorption spectrum with lengthening the gap size. When the nanorod is separated away from the nanofilm, the resonance length becomes longer due to the weak interaction between them^57^. When *d* = 5 *nm*, the resonance length for exciting the 16-pole mode is *a* = 175 *nm*; while for the same resonant mode, *a* = 320 nm when the nanorod is separated by *d* = 30 *nm*. Besides the distance *d* between the nanorod and nanofilm, the radius *r* of the nanorod also affects the GSPs significantly. As shown in Fig. 2e, the resonance length of the 16-pole mode shifts to a longer value as *r* increases, in line with the variation tendency of absorption spectrum for the Ag nanorod in homogeneus medium^58^; For example, the resonance length ranges from 180 *nm*, 241 *nm* to 370 *nm* corresponding to *r* = 10 *nm*, 20 *nm* and 30 *nm* respectively.

### High-contrast switching of the SE

The contrast ratio is generally defined as the ratio between the maximum and the minimum of the emission rate. Previous studies^29–34^ of using the density of optical states to modulate the SE can not achieve high-contrast ratio. For examples, electrically tuning the Fermi level in 2D optical materials can result in a contrast ratio of only 2^30^; changing the optical axis of the LC with plasmon nanofilm, the SE rate can be tuned by a factor of only 2.5 times^39^. For practical applications of quantum devices, our goals here are to obtain simultaneously on a single device, (i) the large enhancement of the SE, (ii) significant contrast ratio, and (iii) high-efficiency routing of emitted photons. In the following, we first explore the switching of the SE in the proposed GSP nanostructure without the nanofiber, where the first two goals can be achieved. The quantum emitter with vertical polarization (oriented along the Z axis) is chosen because in this situation its SE rate is almost two orders of magnitude larger than that with horizontal oriented dipole.

**Figure 3:**
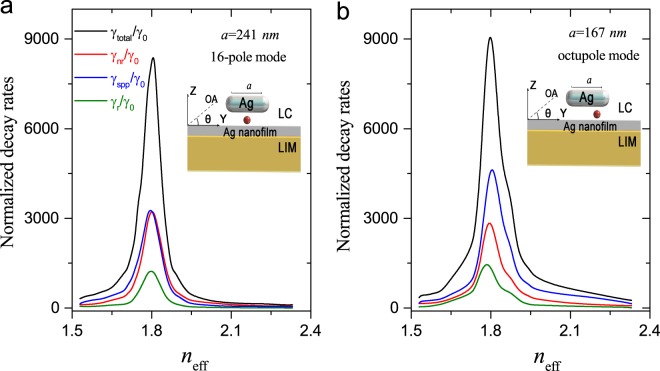
High-contrast switching of the SE. (**a**) Switching SE based on the 16-pole mode as a function of *n*_eff_. Here, γ_total_ can be changed from 103 γ_0_ to 8750 γ_0_ with the contrast ratio of 85, γ_spp_ from 42 γ_0_ to 3726 γ_0_ with the ratio of 88, and γ_nr_ from 60 γ_0_ to 3643 γ_0_ with the ratio of 60. (**b**) Switching SE based on the octupole mode as a function of *n*_eff_. Here, γ_total_ can be changed from 357 γ_0_ to 9287 γ_0_ with the contrast ratio of 26, γ_spp_ from 273γ_0_ to 4856γ_0_ with the ratio of 17, and γ_nr_ from 78 γ_0_ to 3012γ_0_ with the ratio of 38. Compared with the 16-pole mode, in the octupole mode, contrast ratios for all decay rates decrease due to the broader linewidth.

Figure 3a displays the high-contrast switching of the SE rate based on the 16-pole GSP. In this case, the dipole emitter is placed in the middle of the nanogap, which is the position of the brightest hotspot of the 16-pole mode. For *n*_eff_ = 1.81 (or *θ* = 45°), the resonance length of nanorod is *a* = 241 *nm*, as seen in Fig. 2a. In this case, the maximum value of γ_total_ is 8750 γ_0_ due to the efficient excitation of the 16-pole mode. By changing the *n*_eff_ (or varying *θ*), the minimum value of γ_total_ = 103 γ_0_ is arrived at for *n*_eff_ = 2.33 (or *θ* = 90°). Thus we have a contrast ratio of 85, which illustrates the superiority of the present design of the GSP nanostructure over previous works^29–34^. In the switching process, all decay channels change in a similar fashion: γ_spp_ changes from 42 γ_0_ to 3726 γ_0_ with the contrast ratio of 88 (blue curve), γ_nr_ from 60 γ_0_ to 3643 γ_0_ with the ratio of 60 (red curve), and γ_*r*_ can be changed from γ_0_ to 1381 γ_0_ (green curve). Here the most valuable part is γ_spp_ because its extraction into the nanofiber γ_fiber_ (in the following) is very useful in practical applications, such as nanolasers or single photon sources. It is worth noting that γ_total_ varies rapidly with *n*_eff_, i.e., from 800γ_0_ to 7300γ_0_, when *n*_eff_ changes from 1.84 to 1.94. Such small change in LC index can be effected very quickly by several means, approaching the sub-microsecond or nanoseconds time scale as demonstrated in experiments^40^.

As shown in Fig. 3b, high-contrast switching of the SE rate can also be achieved using the octupole GSP. In this case, the quantum emitter is placed at the quarter of the nanoscale gap where one of the brightest hotspots of the octupole mode resides. With the resonance length of *a* = 167 *nm*, it is found that γ_total_ can be changed from 357γ_0_ to 9287γ_0_ with the contrast ratio of 26, γ_spp_ from 273γ_0_ to 4856γ_0_ with the ratio of 17, and γ_nr_ from 78γ_0_ to 3012γ_0_ with the ratio of 38. Compared with the 16-pole mode, γ_spp_ accounts for a large proportion of γ_total_ due to the low metallic loss. But the contrast ratios for all decay channels fall more than half. This may be explained by the difference in the shape of two modes, i.e., the 16-pole mode has a narrower lineshape than the octupole mode. Thus, for the consideration of the high-contrast switching, the 16-pole mode is preferred.

**Figure 4:**
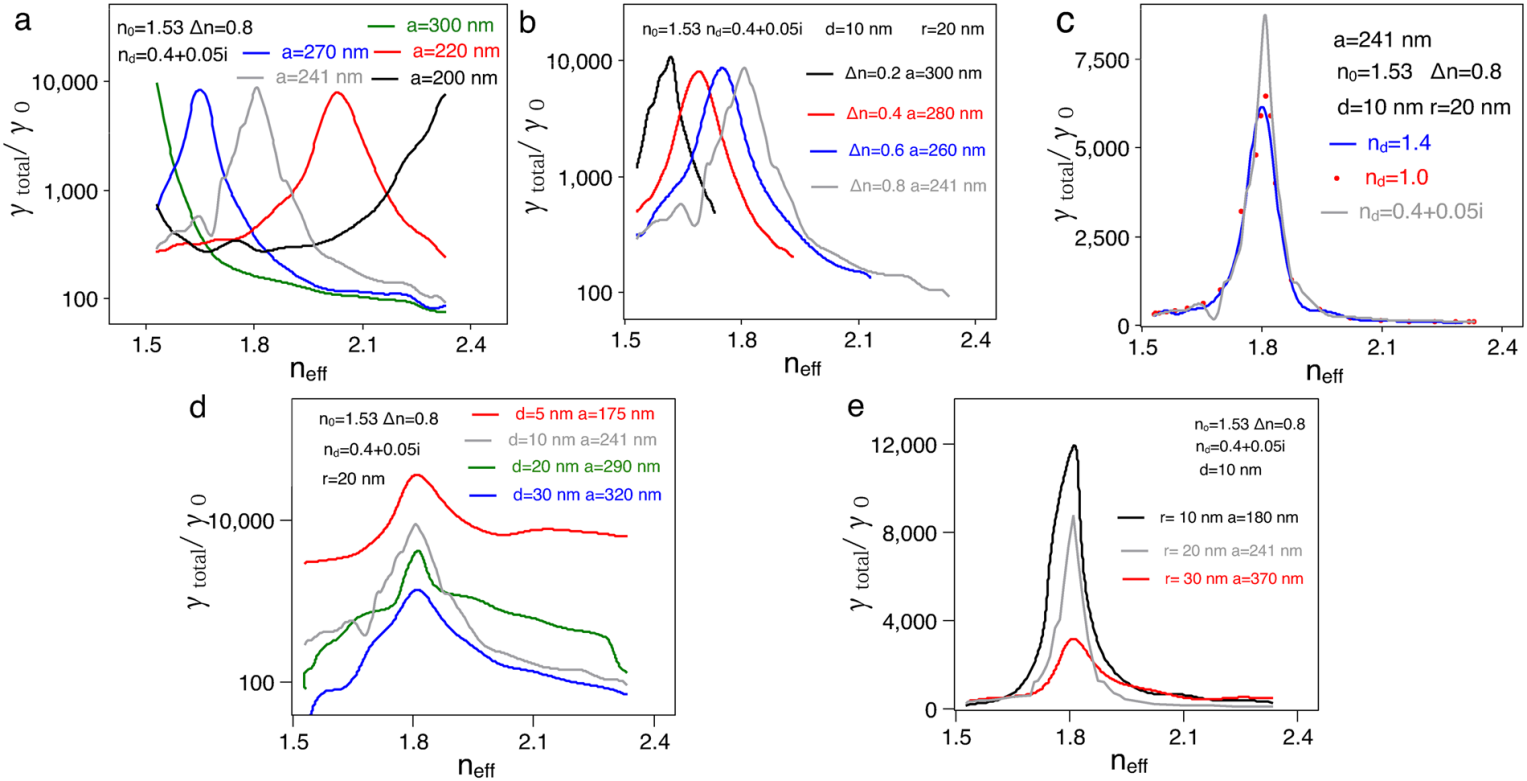
The effects of material and geometric parameters on switching SE for the 16-pole mode. (**a**–**c**) Switching *γ*_total_ for different resonance lengths, different Δ*n* and different *n*_d_. Here, the material parameters are corresponding to the modes shown in Fig. 2a–c and the geometric parameters are the same as those in the Fig. 1b. (**d**,**e**) Switching *γ*_total_ for different *d* and *r*. Here, the geometric parameters are corresponding to the modes shown in Fig. 2d,e and the material parameters are the same as those in the Fig. 1b.

Switching SE strongly depends on the change of the hotspots of the GSPs, which are mainly determined by material and geometrical characteristics. In the above discussions, for example, the 16-pole GSP is resonant at *n*_eff_ = 1.81 with *θ* = 45°, which means the maximum value of the decay rate is obtained at specific effective refractive index of the LC. Now, we further analyze the effects of material and geometrical characteristics on the enhancement of SE rate as well as the contrast ratio of switching SE for the 16-pole mode. Figure 4a displays the switching of *γ*_total_ under different resonance lengths. Here each resonance length corresponds to the mode with fixed optical axis, i.e., a specific permittivity tensor of LC, shown in Fig. 2a. It is seen that the contrast ratio of *γ*_total_ decreases with shortening of the resonance length. For examples, when the resonance length *a* = 300 *nm*, the contrast ratio is 120 for the maximum value of *γ*_total_ = 9521*γ*_0_ with *n*_eff_ = 2.33 and minimal value of *γ*_total_ = 80*γ*_0_ with *n*_eff_ = 1.53; On the other hand, for the resonance length of *a* = 200 *nm*, the contrast ratio is 27 for the maximum value of *γ*_total_ = 7511 *γ*_0_ with *n*_eff_ = 1.53 and minimal value of *γ*_total_ = 277*γ*_0_ with *n*_eff_ = 1.65. Generally speaking, a larger change in the effective refractive index gives rise to a higher contrast ratio of switching due to more drastic changes of the GSPs.

Both the SE rate and its contrast ratio of switching depend on the birefringence Δ*n* of refractive index in the LC and the refractive index *n*_d_ of the LIM. Figure 4b and c depict the switching of the *γ*_total_, where the excitations of GSPs correspond to the modes that are shown in Fig. 2b and c. As shown in Fig. 4b, the contrast ratio becomes higher when Δ*n*. When Δ*n* = 0.2, the contrast ratio is 21 for the maximum value of *γ*_total_ = 10524 and minimal value of *γ*_total_ = 490*γ*_0_; as for Δ*n* = 0.8, the contrast ratio is 85 for the maximum value of *γ*_total_ = 8750*γ*_0_ and minimal value of *γ*_total_ = 103*γ*_0_; The contrast ratio, however, remains almost the same value of 85 for *n*_d_ varying from *n*_d_ = 0.4 + 0.05*i* to *n*_d_ = 1.4; i.e., the refractive index *n*_d_ only has very little effect on the contrast ratio, as shown in Fig. 4c even though the value of *n*_d_ does determine the enhancement of SE rate [Note: the maximum value of *γ*_total_ decreases from 8750 *γ*_0_ to 6000 *γ*_0_ corresponding *n*_d_ = 0.4 + 0.05*i* and *n*_d_ = 1.4].

Other factors such as the nanorod-nanofilm separation *d* and the radius *r* also influence the enhancement of the SE rate and switching contrast ratio. As the nanorod is separated away from the nanofilm, SE rate enhancement decreases due to the lower intensity of GSPs^39^. As shown in Fig. 4d, when *d* = 5 *nm*, γ_total_ changes from 2923γ_0_ to 35846γ_0_ while γ_total_ changes from 21 γ_0_ to 324 γ_0_ when *d* = 40 *nm*. Notably, if the nanorod is too close to the nanofilm (∼2 *nm*), the quantum quenching has to be considered for SE^59^, which is not our main target for SE switching. The data shown in Fig. 4e illustrates the influence of radius *r*. For *r* = 10 *nm*, γ_total_ changes from 184γ_0_ to 11886γ_0_ while γ_total_ changes from 270γ_0_ to 3189γ_0_ for *r* = 30 *nm*. As for the contrast ratio, there exists an optimal set of *r* and *d* that enables both large enhancement of SE rate and high-contrast ratio (See Supplementary Information). These results would provide useful guides for contemplating experiments.

### High-efficiency routing for the spontaneous emission

In the previous section, we demonstrate that the enhancement of SE rate can exceed more than 8000 and its contrast ratio for switching can reach 85 in designed tunable GSP structure. Despite these advantages, this hybrid system suffers from inevitable high metallic losses and low directionality of the emission, which limit the potential applications. For efficiently collecting these photons, one can utilize the optical antennas to couple the emitted photons directly into external optical devices^60,61^. As the quantum emitters are in the vicinity of the metallic and dielectric nanowires, the emitted photons are collected by the evanescent waves and directly guided along the nanowires with high coupling efficiency^35–37^. In previous GSP system^23^, a single phase-matching nanofiber is designed to collect the emitted single photons with a collection efficiency is only about 15%. In order to attain higher efficiency in the collection and low-loss guiding of the emitted photons, we propose here a design that incorporates two symmetrical dielectric nanofibers phase-matched with the surface plasmons of the nanofilm as schematically shown in Fig. 1a.

The double nanofibers are symmetrically placed near the nanorod with the distance of 30 *nm* away from the axle wire of nanorod and 10 *nm* away from the nanofilm. In order to obtain the higher collection efficiency, we choose the rectangle nanofibers with the dimension of 800 × 640 *nm*^2^ rather than cylindrical nanofibers. By considering various semiconductors^62^, the optimized choice is a pair of low-loss phase-matched *Si*_3_*N*_4_ nanofibers with the refractive index of *n* = 2.46. Note that the existence of double nanofibers only has little influence on the decay channels since they are made of dielectric material (See Supplementary Information). Through coupling the evanescent waves of Ag nanofilm to the phase-matched nanofibers, the extraction decay rate γ_fiber_ into nanofibers mainly comes from the decay rate γ_spp_ of surface plasmon. It is believed that the radiative part γ_r_ also has some but small contribution to the γ_fiber_ due to the photon scattering on the nanofibers.

**Figure 5:**
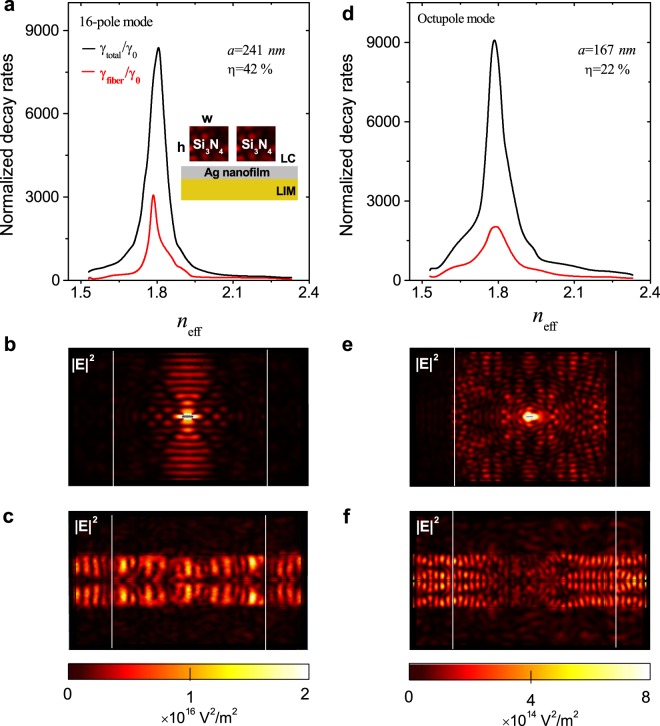
High-efficiency routing for the spontaneously emitted photons. γ_total_/γ_0_ and γ_fiber_/γ_0_ for (**a**) the 16-pole and (**d**) the octupole mode as a function of *n*_eff_. The collection efficiency is about 42% for the 16-pole mode and about 22% for the octupole mode. The insert in (**a**) shows the cross section of double nanofibers with the dimensions of *w* = 800 *nm* and *h* = 640 *nm*. The distributions of electric field intensities without double nanofibers for (**b**) the 16-pole mode and (**e**) octupole mode in the LC layer 80 *nm* above the nanofilm. The distributions of electric field intensities with double nanofibers for (**c**) the 16-pole mode and (**f**) octupole mode in the LC layer 300 *nm* above the nanofilm. The module used here is adjusted to 6 × 4 × 1 *μm*^3^ while the silver film dimension in the XY plane is 4 × 4 *μm*^2^, which is edged by white lines. Other parameters are the same as those in Fig. 1b.

High-efficiency routing of SE based on the 16-pole mode and the octupole mode is shown in Fig. 5. When the dipole emitter is placed at its brightest hotspot with the parameters *n*_eff_ = 1.81 and *a* = 241 *nm*, the 16-pole mode is excited efficiently. As shown in Fig. 5a, with the nanofibers, the high-contrast ratio 90 of switching the emission rate (denoted as γ_fiber_) still remains with the maximum value of 3591γ_0_ for *n*_eff_ = 1.81 and the minimum value of 40γ_0_ for *n*_eff_ = 2.33. Without the nanofibers, the electrical field intensity is concentrated along the direction perpendicular to the long axis of the nanorod in Fig. 5b. When the double nanofibers are symmetrically placed parallel to the long axis of the nanorod, the emitted photons can be efficiently collected with the collection efficiency *η* (=γ_fiber_/γ_total_) of about 42%; the electric field intensities are shown in Fig. 5c.

The octupole mode is excited efficiently when the emitter is at its brightest hotspot with the parameters *n*_eff_ = 1.81 and *a* = 167 *nm*. As shown in Fig. 5d, with the incorporation of nanofibers, the emission rate γ_fiber_ changes from 85γ_0_ to 2030γ_0_ with the contrast ratio of 24 and its collection efficiency *η* is only about 22%. From the distribution of electric field intensity in Fig. 5e and f, one can see that smaller fraction of the emission photons are collected by the nanofibers due to its unidirectional emission angle. Thus, for efficient collecting and guiding of SE for use in controllable on-chip quantum devices, the 16-pole mode is preferred. When the distance *d* between the nanorod and nanofilm increases, the γ_fiber_ also decreases dramatically as previously demonstrated^23^. The collection efficiency *η*, mainly contributed by the evanescent coupling, decreases slightly due to the combined effects of increasing the distance *d* and the resonant length *a*, i.e. there is lower possibility for photons to couple into the nanofibers when the distance increases^63^.

## Discussion and Conclusion

Using current nano fabrication technologies, nanoparticle-coupled nanofilm plasmon structures have been fabricated in isotropic environment where the size of the nanorod, the nanofilm, and the gap distance can be accurately controlled^22^. However, the fabrication of ordered assemblies of nanoparticles in anisotropic LCs remains a challenging issue due to additional elasticity-mediated interaction. Fortunately, there has been recent breakthrough in this area, i.e., the gold nanorod is directly fabricated in a nematic LC^64^, indicating feasibility of our design that allows tunability by changing the refractive index of the LC. There are so many ways to change the refractive index of LC besides modulating its optical axis, e.g., it can be changed by light through a variety of nonlinear optical mechanisms or simply by temperature/order parameter modification^40^. Various efforts have been made to fabricate metamaterials with novel refractive index values ranging from positive, through zero, and into the negative domain^52^. Recently, the fishnet metamaterials containing the LC and gold particles have been also reported^65^. The low-loss dielectric nanofiber for routing photons has been fabricated by various methods including chemical synthesis and laser ablation^66^. Inserting a single dipole emitter into the structure is clearly a monumentally difficult task, but considerable insights can be gained by examining the fluorescence enhancement of spatially independent single emitter in a dilute layer of fluorescent molecules coated on the structure^67^. All these suggest that, using the present state-of-the-art techniques, it will be feasible to experimentally realize our proposed nanostructures in the near future.

In conclusion, we have demonstrated high-contrast switching of fluorescence intensity as well as high-efficient routing of emission photons in our designed tunable GSP design. The switching performance, controlled by the material parameters of the LC and low index materials while the custom-designed symmetrical double nanofibers, greatly improve the efficiency for collection and channeling of photons. With these unique advantages, our results will likely contribute to the development of highly desirable ultra-compact quantum photonic devices for quantum computation and quantum information processing.

## Methods

### The setup of simulation module

Numerical simulations of the GSPs and the SE rates are performed by the commercial COMSOL Multiphysics software. To better approximate the behavior of an infinitely extended metal film, the geometry of module is chosen as 4 × 4 × 1 *μm*^3^, and a perfectly matched layer of 200 *nm* is introduced to minimize boundary reflections. The thickness of LC, Ag nanofilm, and LIM layers are 750 *nm*, 50 *nm* and 200 *nm* respectively. To more closely approximate the experimental conditions and avoid numerical inaccuracy, an additional air layer of 50 *nm* covers on the LC layer. Finally, the quantum emitter (at the hotspots) oriented along the Z axis is chosen because its total decay rate is several dozen times larger than that of X or Y axis oriented dipole emitters. Using the similar module, we have studied the nanoparticle surface plasmon resonance images of electrocatalytic activity^68^, and the efficient emission of single photons and reversible photon-exciton interaction in the GSPs nanostructures^23,69^.

### The excitation of the GSPs

There are two methods to excite the GSPs. One is to use the background field to excite the GSPs, where the polarization of the light is parallel to Y axis. To avoid reflections owing to truncating the simulation space, one can use the scattered field formulation where the background field is specified using the plane wave. Another is to locally drive the GSPs by the point dipole emitter. Under the full field formulation, the dipole emitter placed at the end of the nanorod can break the symmetry of hotspots, thus exciting all possible GSPs. To obtain the spectra of the GSPs, the resistive loss as a function of the length of Ag nanorod is calculated by performing the volume integral for the Joule heating within nanorod. Both the methods can excite the GSP with same resonant spectrum. In the section B of the Results, to sufficiently excite all possible modes, we use the second method, i.e., a dipole emitter with Y axis polarization is set at 5 *nm* away from the end of nanorod.

### Calculation of decay rates

Here, we give the computation details for all decay rates of the dipole emitter. The emitter decays through three channels^23^: surface plasmon channel with decay rate γ_spp_, far field channel with decay rate γ_*r*_, and nonradiative channel with γ_nr_. By considering all the possible decay channels of dipole emitter, γ_spp_ can be obtained by eliminating all the other decay rates from the total decay rate, i.e. γ_spp_ = γ_total_-γ_nr_-γ_r_. Thus normalized decay rate into surface plasmon channel is:

$$\frac{{{\rm{\gamma }}}_{{\rm{spp}}}}{{{\rm{\gamma }}}_{0}}=\frac{{P}_{{\rm{total}}}-{P}_{{\rm{nr}}}-{P}_{{\rm{r}}}}{{P}_{0}}$$

where *P*_total_ is the dipole radiative power obtained by performing surface integrals over the Poynting vector *S* on the 4 *nm* radius sphere. The nonradiative power *P*_nr_, including the absorption of both metallic nanorod and nanofilm, can be obtained by performing the volume integral for the Joule heating in the area of nanorod and its image in the nanofilm^23,39^. Meanwhile, by performing surface integrals over the Poyning vector *S*_*z*_ on the upper and lower boundaries of the model, the radiative power *P*_r_ can be obtained. As for the routing part *γ*_fiber_ of SE, the module used here is adjusted to 6 × 4 × 1 *μm*^3^, the silver film dimension in the XY plane is 4 × 4 *μm*^2^ to avoid interactions between the nanofiber and the nanofilm. The cross-sectional dimension of nanofiber is 800 × 640 *nm*^2^ and its length is set as 6 *μm*. γ_fiber_ is calculated by performing surface integrals over the Poynting vector *S*_*x*_ on the fiber cross section 2 *μm* away from the emitter.

## Electronic supplementary material

Supplementary Information
