# Supplementary material for: High-contrast switching and high-efficiency extracting for spontaneous emission based on tunable gap surface plasmon
Source: Sci Rep. 2018 Jul 26;8:11244. doi: 10.1038/s41598-018-29624-y (PMC6062572; doi:10.1038/s41598-018-29624-y)
Supplement: Supplementary file 1 — Supplementary Information [file 41598_2018_29624_MOESM1_ESM.pdf]

Supplementary Information

High-contrast switching and high-efficiency extracting for spontaneous emission based on tunable gap surface plasmon

He Hao,[1] Juanjuan Ren,[1] Xueke Duan,[1] Guowei Lu,[1,2] Iam Choon Khoo,[3] Qihuang Gong,[1,2] and Ying Gu[1,2,a)]

[1)] *State Key Laboratory for Mesoscopic Physics, Collaborative Innovation Center of Quantum Matter, School of Physics, Peking University, Beijing, 100871, China*

[2)] *Collaborative Innovation Center of Extreme Optics, Shanxi University, Taiyuan, Shanxi, 020006, China*

[3)] *Department of Electrical Engineering, 121 Electrical Engineering East, Pennsylvania State University, University Park, PA 16802, USA*

(Dated: 18 June 2018)

[a)] Electronic mail: ygu@pku.edu.cn

1. Geometrical Effects on the GSPs and Switching SE

1.1 The effect of the size of Ag nanorod on the GSPs and switching SE

The spectral properties of the GSPs depend on not only the material characteristics but also the geometrical factors, such as the size of nanorod, the distance between nanorod and nanofilm[1–3]. The properties of the GSPs and the performance of switching SE vary strongly with the radius r. As shown in Fig. S1, when r decreases, the resonance length for each multipole mode shifts to a longer value, in line with the variation tendency of absorption spectra for the Ag nanorod in homogeneus medium[1]. For example, the resonance length for the 16-pole mode changes from 180 nm, 241 nm to 370 nm corresponding to r=10 nm, 20 nm and 30 nm respectively. When the radius is smaller, the higher γ_{total} will be obtained. For r=10 nm, γ_{total} switches from 184γ_0 to 11886γ_0 [contrast ratio of 64], while γ_{spp} changes from 14γ_0 to 2358γ_0 [contrast ratio of 168]; whereas for r=30 nm, γ_{total} changes from 270γ_0 to 3189γ_0 [contrast ratio of 12], and γ_{spp} changes from 138γ_0 to 2128γ_0 [contrast ratio of 15]. However, such large contrast ratios are accompanied by considerable rise in the non-radiative component γ_{nr} and therefore a lower fraction of γ_{spp} in the total decay rate. When r=10 nm, γ_{nr} changes from 170γ_0 to 8862γ_0, occupying more than 75% of γ_{total}=11886γ_0, compared to the case for r=30 nm where the fraction of γ_{nr} (changing from 122γ_0 to 674γ_0) in the total decay rate γ_{total}=3189 is less. Therefore, in the discussion presented in the main text, r=20 nm is chosen to balance the enhancement of SE rate and the metallic loss.

1.2 The effect of the distance between nanorod and nanofilm on the GSPs and SE modulation

A parameter that produces more dramatic effects on the GSPs and SE modulation is the gap distance d between the nanorod and nanofilm[2,3]. The data shown in Fig. S2 illustrate the trend of absorption spectrum with lengthening the gap size. When the nanorod and the nanofilm are further apart, the resonance length becomes longer due to the weaker interaction between them. For d=5 nm, the 16-pole mode shows a resonance at about a=175 nm, while for d = 40 nm, the resonance length a=340 nm. Besides, owing to the ultra-concentrated field for the small gap size, all decay rates experience the large enhancement, but modest contrast ratio. For example, when d=5 nm, γ_{total} changes from 2923γ_0 to 35846γ_0 with the contrast ratio of only 12, and γ_{spp} changes from 2790γ_0 to 19579γ_0 with the contrast ratio of only 7. When the gap size becomes larger, all decay rates decreases, e.g., when d=40 nm,

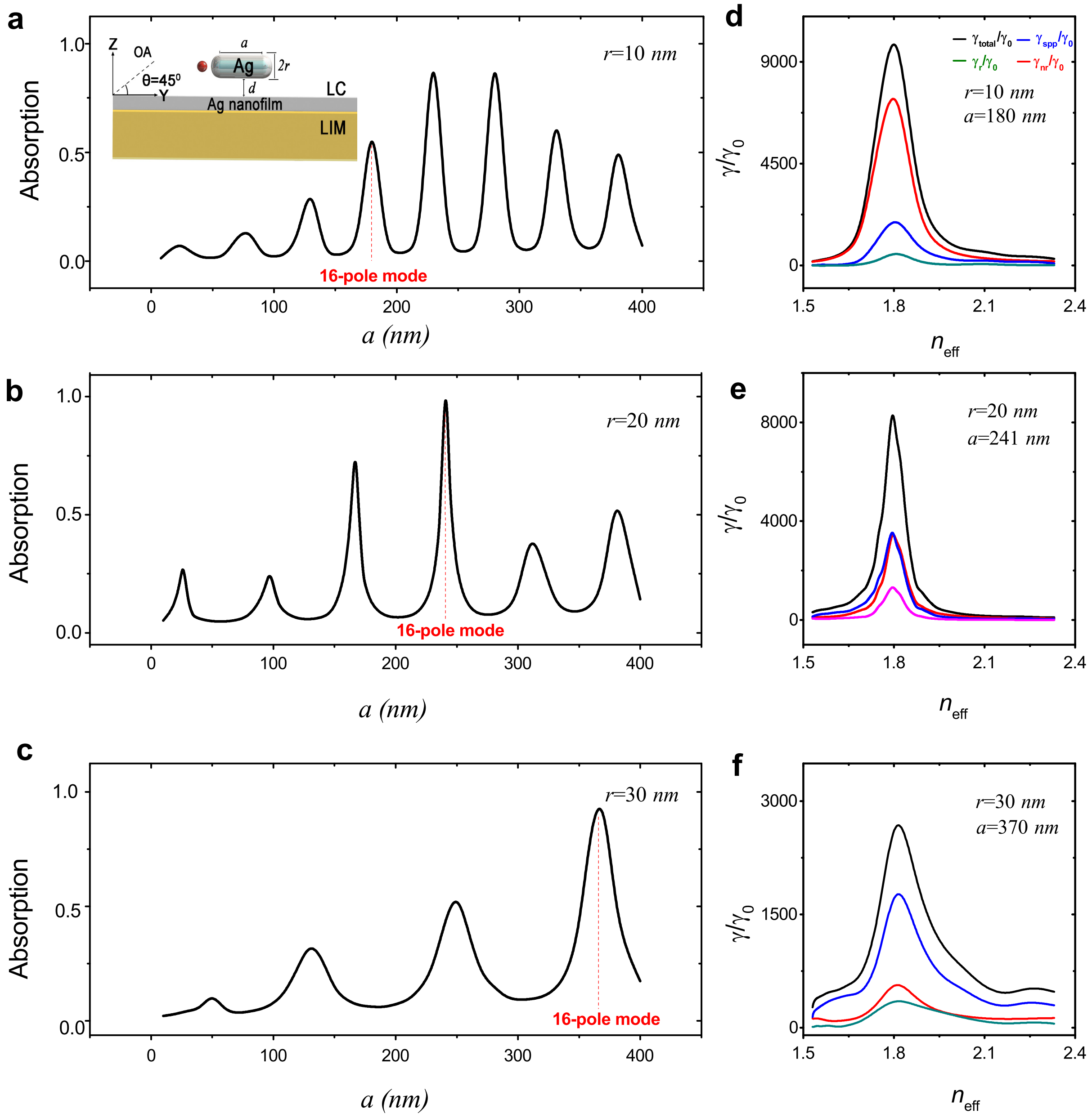

FIG. S1. The effects of the size of nanorod on the GSPs and switching SE. (a)-(c) The absorption spectra of the GSPs with varying the radius r of nanorod. The resonance length shifts to longer values with the increase of r. The single emitter at the end of the nanorod is to guarantee that all possible modes can be excited efficiently. (d)-(f) SE modulation based on the 16-pole mode for different r. The resonance lengths correspond to the 16-pole modes shown in the (a)-(c). Here the emitter is placed at the middle of the nanogap, which is the position of the brightest hotspot of the 16-pole mode. The enhancement of the SE rate decreases with the increase of r while the highest contrast ratio of switching SE is obtained when r=20 nm. Other parameters are the same as those in Fig. 2a of the main text.

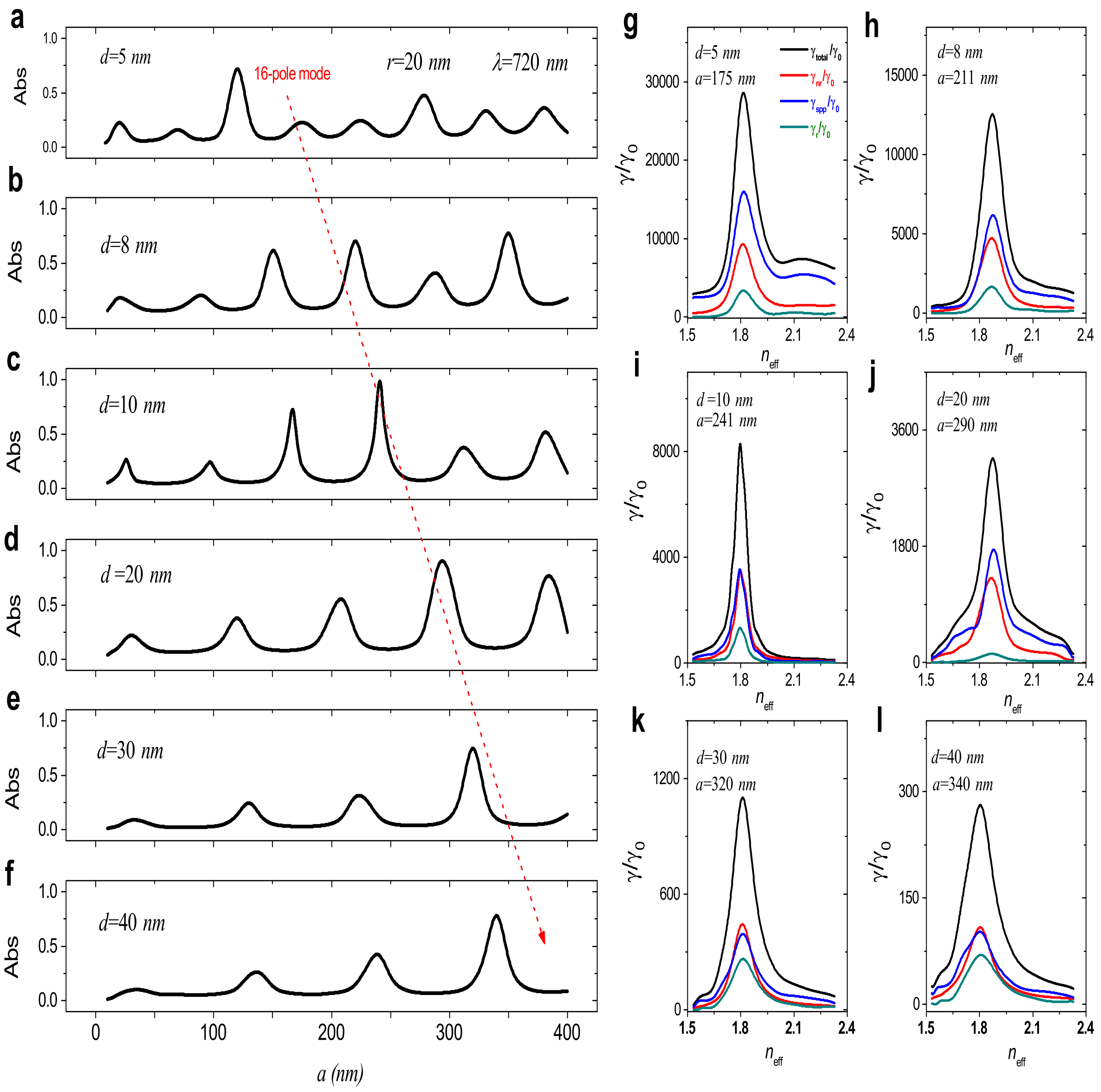

FIG. S2. The effects of the distance d between nanorod and nanofilm on the GSPs and switching SE. (a)-(f) The absorption spectra of GSPs with varying the distance d. The resonance length becomes longer when the gap distance d increases. (g)-(l) SE modulation based on the 16-pole mode for different d. The enhancement of SE rate decreases with the increase of the d while the highest contrast ratio of switching SE is obtained when d=10 nm.

γ_{total} changes from $21\gamma_0$ to $324\gamma_0$ with the contrast ratio of 15, and γ_{spp} changes from $9\gamma_0$ to $116\gamma_0$ with the contrast ratio of 12. As for the contrast ratio, the variation of r or d would not significantly influence both the maximum and minimum value of γ_{total}, i.e. there is no

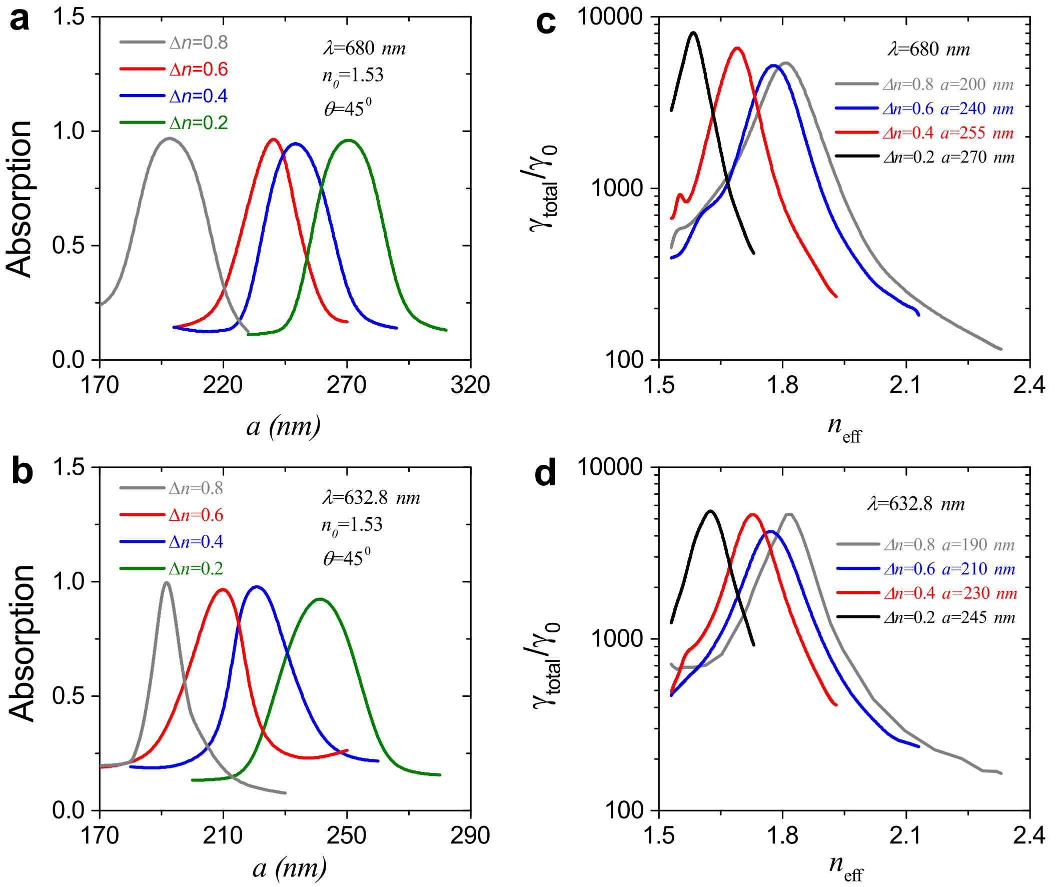

FIG. S3. The effects of wavelength on the GSPs and switching SE. The absorption spectra of the 16-pole mode for (a) λ=680 *nm* and (b) λ=632.8 *nm*. The resonance length becomes longer when the wavelength red shifts. Switching γ_{total} based on the 16-pole mode for (c) λ=680 *nm* and (d) λ=632.8 *nm*. The contrast ratio is higher for longer wavelength. Other parameters are the same as those in the Fig. 4a of the main text.

direct relationship between the contrast ratio and r or d. There exists however a optimal set of r and d for both large enhancement of SE rate and high-contrast ratio. As shown in the main text, when r=20 *nm* and d=10 *nm*, the contrast ratio can reach about 85 with the enhancement of SE rate of $8750\gamma_0$.

2. Effects of the wavelength on the GSPs and switching SE

In the main text, we demonstrate both the large enhancement of SE rates and high-contrast ratio of SE switching for a particular wavelength λ=720 *nm*. These findings are actually also valid across a broad range of emission wavelength[4]. Fig. S3 shows the properties of GSPs and the switching of SE based on 16-pole mode at different wavelengthes. No matter

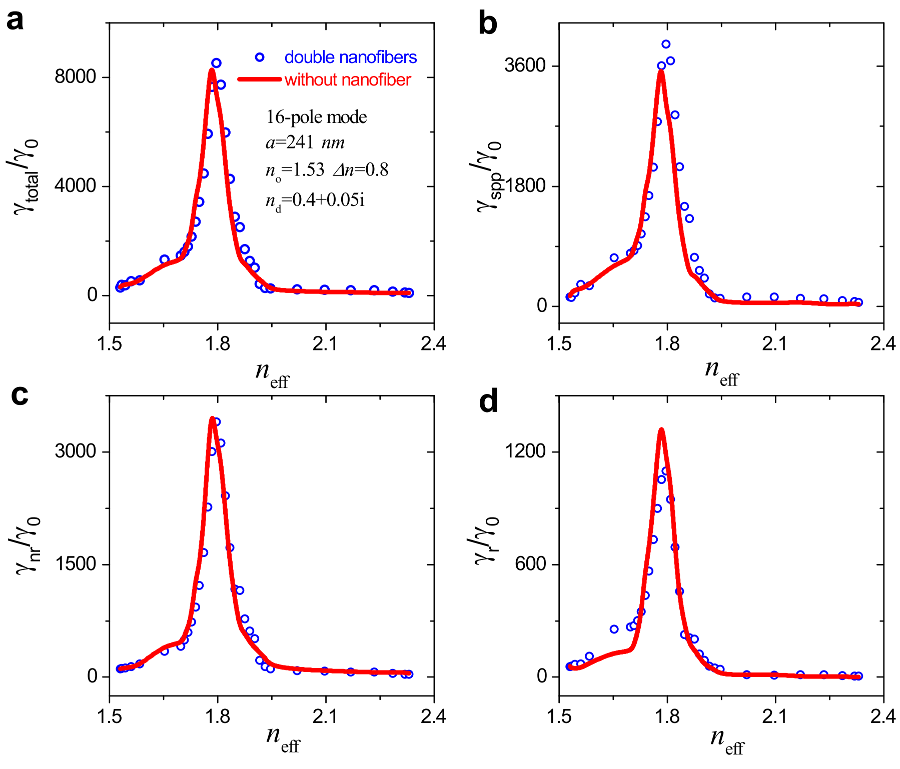

FIG. S4. The influences of double rectangle nanofibers on switching SE. Decay rates (a) γ_{total}, (b) γ_{spp}, (c) γ_{nr} and (d) γ_{r} as a function of n_{eff} with and without nanofibers. The nanofibers has little influence on the SE modulation. Here, the parameters are the same as those in Fig. 4a of the main text.

for λ=680 nm or λ=632.8 nm, the resonance length for the 16-pole mode shifts to longer value when Δn decreases, in line with the results obtained at λ=720 nm (Fig. 3 in the main text). The dependence of the contrast ratio on the Δn also remains the same as for λ=720 nm (Fig. 5 in the main text), i.e., the contrast ratio becomes higher when Δn increases.

Besides, under the same parameters, the resonance length for the same order of GSPs at longer wavelength shifts to longer value. For the example of the 16-pole mode at Δn=0.8, the resonance length a=190 nm for λ=632.8 nm (Fig. S3b) while a=200 nm for λ=680 nm (Fig. S3a). For λ=720 nm, the resonance length increases to a=241 nm (Fig. 5a in the main text). As for the contrast ratio, as λ=632.8 nm, γ_{total} can change from 164γ_0 to 5318γ_0 with the contrast ratio of 32 at Δn=0.8 (Fig. S3d). For the same Δn with $\lambda = 680$ nm, γ_{total} changes from 115γ_0 to 7280γ_0 with the contrast ratio of 63 (Fig. S3c). Both the maximum decay rate and the contrast ratio increases when the wavelength red shifts,

reaching the value of $8750\gamma_0$ with the contrast of 85 for λ=720 nm. These dependencies can be attributed to the change of the resonance length, in line with what we demonstrated in Fig. 5a of the main text.

3. Influence of double rectangle nanofibers on switching SE

To efficiently collect and guide the emitted photons, we design the symmetrical double dielectric rectangle nanofibers in such a tunable gap plasmon nano-structure. The double nanofibers have little influences on all decay rates since they are made of dielectric material. As shown in the Fig. S4, we display all decay rates with and without double nanofibers. For $\gamma_{\rm nr}$, the two sets of data coincidence with each other very well. For $\gamma_{\rm spp}$ and $\gamma_{\rm r}$, the values obtained with and without nanofibers agree well except near $n_{\rm eff}$=1.65 and $n_{\rm eff}$=1.81. In the case of $n_{\rm eff}$=1.81, $\gamma_{\rm spp}$ with double nanofibers is larger than the value obtained without the nanofibers. While $\gamma_{\rm r}$ with the nanofibers is smaller than the values without the nanofibers. Thus, the values of $\gamma_{\rm total}$ obtained without and with the nanofibers agree well with each other. In the case of $n_{\rm eff}$=1.65, the values of $\gamma_{\rm r}$ are different in the two situations, but the difference has little contribution to $\gamma_{\rm total}$ since $\gamma_{\rm r}$ occupies a small fraction of the total. Therefore, we can conclude that the double rectangle nanofibers have little influences on the decay rates, and serve well as an efficient routing of the emitted photons to the preferred channel.

4. Collecting photons via different types of nanofibers

In the main text, we choose the nanofibers with rectangle cross section rather than cylindrical nanofiber. This is based on the calculation of the collection efficiencies for both cases. The results are shown in the Table. S1. The collection efficiency for a single nanofiber with circle cross section (with the radius of 350 nm) is 9.8% while it increases to 16.5% for a single rectangle nanofiber with dimension of 800×640 nm^2. Noting that the single nanofiber is not the ideal choice, therefore, we design the symmetrical double nanofibers. In that case, the double circle nanofibers yield a collection efficiency of only 14% whereas the collection efficiency increases to 42% with the double rectangle nanofibers

TABLE S1. Collection efficiencies with different kinds of nanofibers

Type of nanofiber	Collection efficiency
single cylindrical nanofiber (radius=350 nm)	9.8%
single rectangle nanofiber (800 $\times$ 640 nm^2)	16.5%
double cylindrical nanofibers	14%
double rectangle nanofibers	42%

REFERENCES

[1]Link, S. & EI-Sayed. M.A Spectral properties and relaxation dynamics of surface plasmon electronic oscillations in gold and silver nanodots and nanorods. *J. Phys. Chem. B* **103**, 8410–8426 (1999).

[2]Hang, L. et. al. Efficient single photon emission and collection based on excitation of gap surface plasmons. *Phys. Rev. Lett.* **114**, 193002 (2015).

[3]Leveque, G. & Martin, Olivier J. F. Optical interactions in a plasmonic particle coupled to a metallic film. *Opt. Express* **14**, 9971–9981 (2006).

[4]Hao. H. et al. Enhanced modulation of spontaneous emission via plasmonic waveguide cladded with liquid crystal and low index metamaterial. *Opt. Express* **25**, 3433–3444 (2017).
